# Implant Renal Injury‐Responsive Cells to Supplement Erythropoietin and Protect Kidney Injury

**DOI:** 10.1002/mco2.70438

**Published:** 2025-10-30

**Authors:** Hao Nie, Chen Liang, Yingxin He, Siyu Wang, Xiaopeng Zhang, Jun Duan, Jieli Huang, Chen Yu, Yujia Wang, Zixian Zhao, Wei Zuo, Ting Zhang

**Affiliations:** ^1^ State Key Laboratory of Cardiovascular Diseases and Medical Innovation Center, Shanghai East Hospital, School of Medicine Tongji University Shanghai China; ^2^ Department of Nephrology, Tongji Hospital, School of Medicine Tongji University Shanghai China; ^3^ Super Organ R&D Center Regend Therapeutics Shanghai China; ^4^ Department of Pharmacy National University of Singapore Singapore Singapore; ^5^ Kiangnan Institute of Stem Cell Hangzhou China

## Abstract

Anemia poses a life‐threatening risk to individuals with chronic kidney diseases (CKDs). Insufficient production of erythropoietin (EPO) from the injured kidney is the major reason for anemia, while lack of EPO would further aggravate the kidney injury and make a “vicious cycle.” In this study, we dissected the change of endogenous EPO signaling in the injured kidney by spatial transcriptomic analysis and validated the dual beneficial effects of local recombinant EPO administration on both anemia correction and kidney protection. Next, we constructed an injury‐responsive EPO‐producing (iREP) element to sense the kidney damage signal and drive the synthesis and secretion of native EPO. After intrarenal implantation of iREP‐engineered HEK–293T embryonic kidney cells, the mouse kidney would automatically produce more EPO when sensing injury signal, which in turn enhanced red blood cell count and protected the kidney from further injury. Moreover, we cloned urine‐derived SOX9+ epithelial cells (USCs) from healthy donors. The USCs can be transplanted into mouse kidneys, which makes them an alternative candidate cell for iREP engineering. Altogether, the current work proposed a potential approach based on an engineered “smart” cell to treat CKDs.

## Graphical abstract

Insufficient erythropoietin (EPO) production in injured kidneys leads to anemia and further exacerbates kidney damage. We constructed an injury‐responsive EPO‐producing (iREP) element that senses signals of kidney damage and drives the synthesis and secretion of native EPO. After intrarenal implantation of iREP‐engineered cells, the kidney produces more EPO in response to injury signals, thereby protecting itself from further damage.

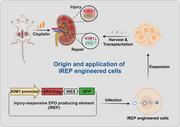

## 1 Introduction

Anemia is a complication of chronic kidney diseases (CKDs) that contributes to multiple adverse clinical outcomes such as the development of left ventricular hypertrophy, progression of kidney disease, decreased quality of life, increased risk of hospitalization, and mortality. This complication was present in 47.7% of 5222 predialysis patients with CKDs and its prevalence increased as kidney function decreased [1]. Many factors could contribute to anemia as CKD progresses, but impaired production of erythropoietin (EPO) due to kidney tissue injury is the central cause [2].

In adults, EPO is a hormone mainly synthesized in the kidney and it is responsible for red blood cell maturation in the bone marrow [3, 4]. In normal status, EPO synthesis is transcriptionally regulated by the oxygen level of the body; while in patients with various advanced kidney diseases, the EPO synthesis process can be largely disrupted, which therefore requires supplement with recombinant analogues [5]. Since the approval of recombinant human EPO by the United States Food and Drug Administration in 1989, epoetin alfa and similar agents now collectively known as erythropoiesis‐stimulating agents (ESA) have become the standard of care for the treatment of anemia that occurs in patients with CKDs [6, 7, 8]. Of note, some observations indicated that ESA treatment demonstrates not only a preventive effect on cardiovascular risks, but also a certain level of protective effect against renal damage [9, 10, 11, 12, 13, 14].

However, recent prospective trials both in the early and late stages of CKD failed to confirm the beneficial effects of ESA on renal damage [6]. Therefore, the effect of recombinant EPO on renal protection is still a subject of debate.

In addition to this uncertainty, the current clinical application of ESA involves the circulation route via subcutaneous or intravenous (I.V.) injections, which demonstrates several shortcomings. First, the commercial non‐native EPO products exhibited a different glycosylation pattern from native ones, which would elicit neutralizing antibodies and even lead to severe pure red‐cell aplasia [15, 16]. Second, by conventional methods, achieving sufficient ESA concentration for local renal protection is challenging. Third, conventional ESA are characterized by shorter half‐lives and limited dosage for each injection, which therefore requires frequent hospital visits and high medical costs [17]. Altogether, the conventional administration of non‐native EPO involves limited renal distribution, substantial costs, and may lead to severe adverse effects, which encouraged urgent need for development of new ESA treatment strategies [18].

To overcome these shortcomings, in the current work, we designed a therapeutic strategy based on the implantation of engineered EPO‐producing cells. By intrarenal implantation of the injury‐responsive EPO‐producing (iREP) cells, functional EPO could be released into the kidney of injured mice to correct anemia and protect the kidney.

## 2 Results

### 2.1 Kidney Local EPO Supplement Demonstrated Both Anemia Correction and Renal Protection Effect

**FIGURE 1:**
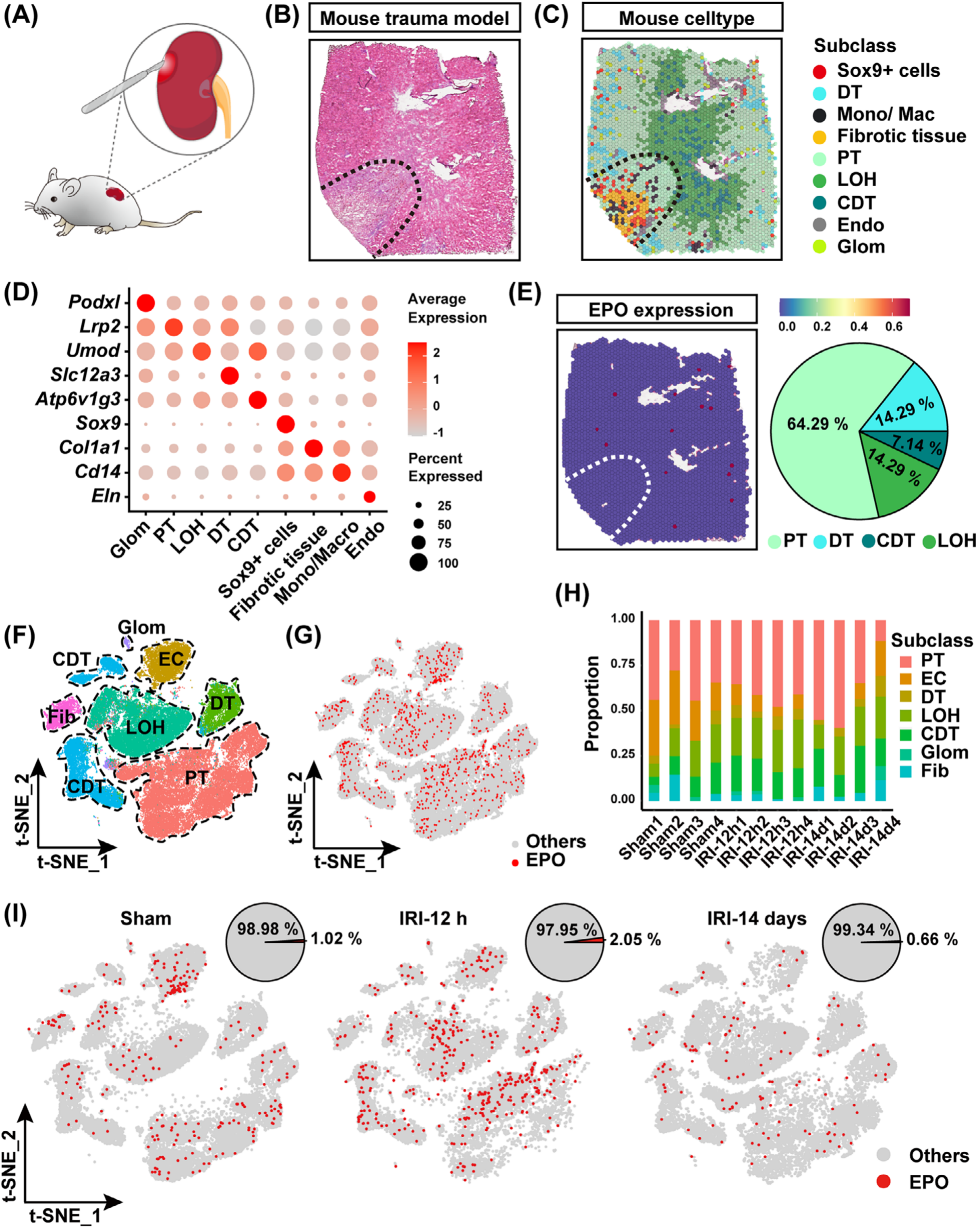
Dynamics of EPO expression in acute kidney injury progression. (A) Schematic diagram of the mouse trauma model using a surgical blade. (B) H&E staining of the mouse trauma model. (C) Spatial transcriptomic mapping of the nine clusters onto the injured mouse kidney. *Abbreviations*: PT, proximal tubule; LOH, Loop of Henle; DT, distal tubule; CDT, collecting duct tubule; Mono/Mac, monocyte/macrophage; Endo, endothelial cells. (D) Dot plot depicting the expression profile of specific genes across various cell types. (E) Expression profile of EPO gene in the spatial transcriptomic map. (F) t‐Distributed Stochastic Neighbor Embedding (t‐SNE) visualization of kidney fractions from a mouse AKI dataset (GSE139107). (G) t‐SNE visualization of EPO gene expression. (H) Cumulative analysis representing the frequency and distribution of EPO+ cells across different individuals. (I) t‐SNE visualization coupled with quantification of EPO+ cells in kidneys from sham control mice (sham, *n* = 4), mice subjected to IRI injury at 12 hours (IRI‐12 hours, *n* = 4), and mice subjected to IRI injury at 14 days (IRI‐14 days, *n* = 4).

To study the function of the EPO signaling pathway in the renal injury process, first we established a mouse model of kidney trauma injury. In this model, a surgical incision on the kidney was made to generate a localized injury area, leaving the neighboring area relatively unaffected (Figure 1A). One week after the injury, pathological sections revealed the loss of normal kidney structure in the incision area, coinciding with characteristic features of inflammatory cell infiltration and tissue fibrosis (Figure 1B). Spatial transcriptome analysis of the mouse kidney showed that the injured area lost many glomerular and renal tubular cells. Instead, there were widespread inflammatory Cd14+ monocytes/macrophages and Col1a1+ fibroblastic tissues in the injured area. Of note, there were some renal tissues showing expression of repair‐related marker Sox9 [19, 20, 21], which were distributed not only within the injured area but also sporadically in the noninjured area (Figure 1C,D).

In adults, the kidney is known as the major organ to secret EPO [4]. Based on the spatial transcriptomic data, here we analyzed the spatial distribution of EPO in the normal and injured areas of the mouse kidney. The results showed that EPO was exclusively expressed in the normal area of the mouse kidney but not in the injured area, and their distribution was mainly within proximal tubular tissue (Figure 1E). Additionally, we analyzed single‐cell RNA sequencing data of the mouse kidneys from the public database (GSE139107) to analyze the expression pattern of EPO. The results showed that EPO was expressed in various cell types of kidney epithelium and endothelium, with the proximal tubules exhibiting the highest proportion (Figure 1F–H). In the mouse kidney ischemia–reperfusion injury (IRI) model, although a rapid increase in EPO expression was observed in the short term (12 hours), over a longer time range (14 days), EPO expression decreased in the injured kidney (Figure 1I). Altogether these data indicated that sub‐acute (or chronic) kidney injury would lead to deficient EPO production, which partly explained the clinically observed EPO deficiency in CKD patients [22, 23].

**FIGURE 2:**
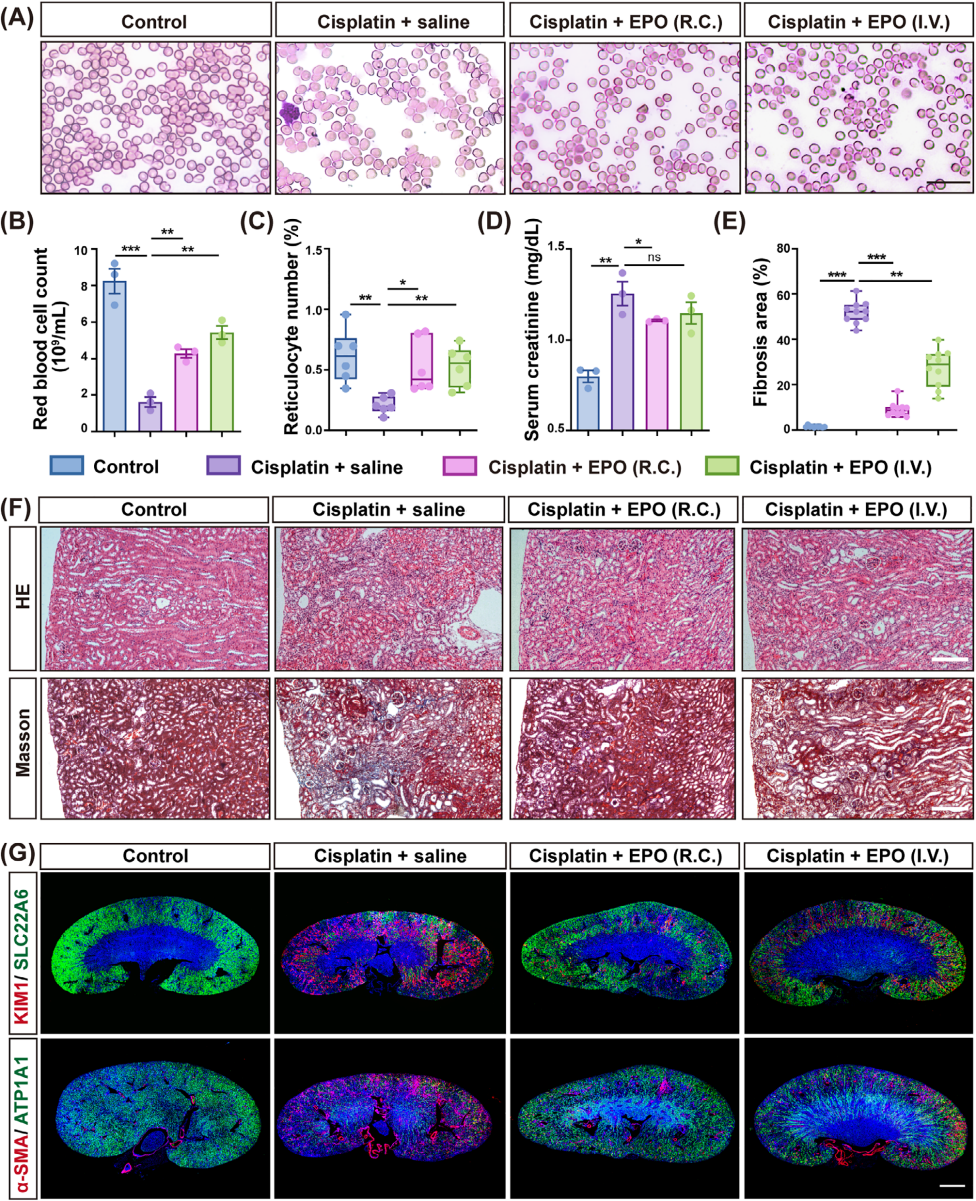
Local EPO supplementation demonstrated renal protection effects. (A) Representative images of the morphology of red blood cells in each group. Scale bar = 20 µm. (B) Comparative quantitative of red blood cell count in cisplatin‐injured mice receiving recombinant EPO versus saline‐treated mice. Control mice received no cisplatin injury. *n* = 3 biological replicates in each group. (C) Percentage of reticulocyte numbers assessed by Brilliant Cresyl Blue staining. *n *= 6 different fields of view in each group. (D) Analysis of serum creatinine. *n *= 3 biological replicates in each group. (E) Quantitation of tubulointerstitial fibrosis in Masson's Trichrome‐stained sections. *n* = 10 different fields of view in each group. (F) Histopathological assessment of mouse recombinant EPO administration in cisplatin‐injured mice. Mice were sacrificed 10 days posttreatment, and kidneys were stained for H&E and Masson's trichrome. Scale bar = 100 µm. (G) Immunofluorescence analysis of normal (SLC22A6, ATP1A1) and injured (KIM1 and α‐SMA) tubular markers. Scale bar = 1 mm. Each group consisted of five mice. Data are shown as mean ± SD. *p < 0.05, **p < 0.01, ***p < 0.001, ns, no significant.

To evaluate the function of EPO on anemia and renal protection, we generated a mouse anemia model based on kidney injury by cisplatin injection, and then tested the effect of recombinant mouse EPO administration. For comparison, a 300 IU/kg EPO dosage was given through either renal capsule (R.C.) or I.V. injection. The result showed that cisplatin induced a significant reduction in red blood cell count, which could be rescued by either R.C. or I.V. EPO injection (Figure 2A,B). Similarly, cisplatin led to a significant decrease in reticulocyte number in peripheral blood, which indicated impaired hematopoiesis. Either R.C. or I.V. EPO administration demonstrated a similar rescue of the reticulocyte number (Figure 2C).

Moreover, cisplatin treatment also led to an increase in serum creatinine levels, which indicated impaired renal function. Interestingly, here we found that only R.C. injection, but not I.V. injection of EPO, could significantly decrease the mouse creatinine levels (Figure 2D). Consistently, hematoxylin and eosin (H&E) staining of kidney sections showed that only R.C. EPO administration remarkably alleviated the renal tubule necrosis and tubulointerstitial area. Masson's trichrome staining showed that although both I.V. and R.C. injection of EPO could reduce the area of renal fibrotic lesion, yet the latter demonstrated significantly better rescue effect (Figures 2E,F). Immunostaining of kidney sections showed more abundant expression of the renal injury marker kidney injury molecule‐1 (KIM1) and myofibroblast marker alpha‐smooth muscle actin (a‐SMA), and less abundant expression of renal tubule markers ATPase Na+/K+ transporting subunit alpha 1 (ATP1A1) and solute carrier family 22 member 6 (SLC22A6) in the cisplatin‐injured kidneys, and only R.C. EPO treatment could alleviate the level of kidney damage (Figures 2G and S1A). Altogether the above data showed that administration of EPO through both systemic and local routes could effectively correct cisplatin‐induced anemia; however, only EPO given through renal local injection could efficiently protect the kidney from severe cisplatin injury.

**FIGURE 3:**
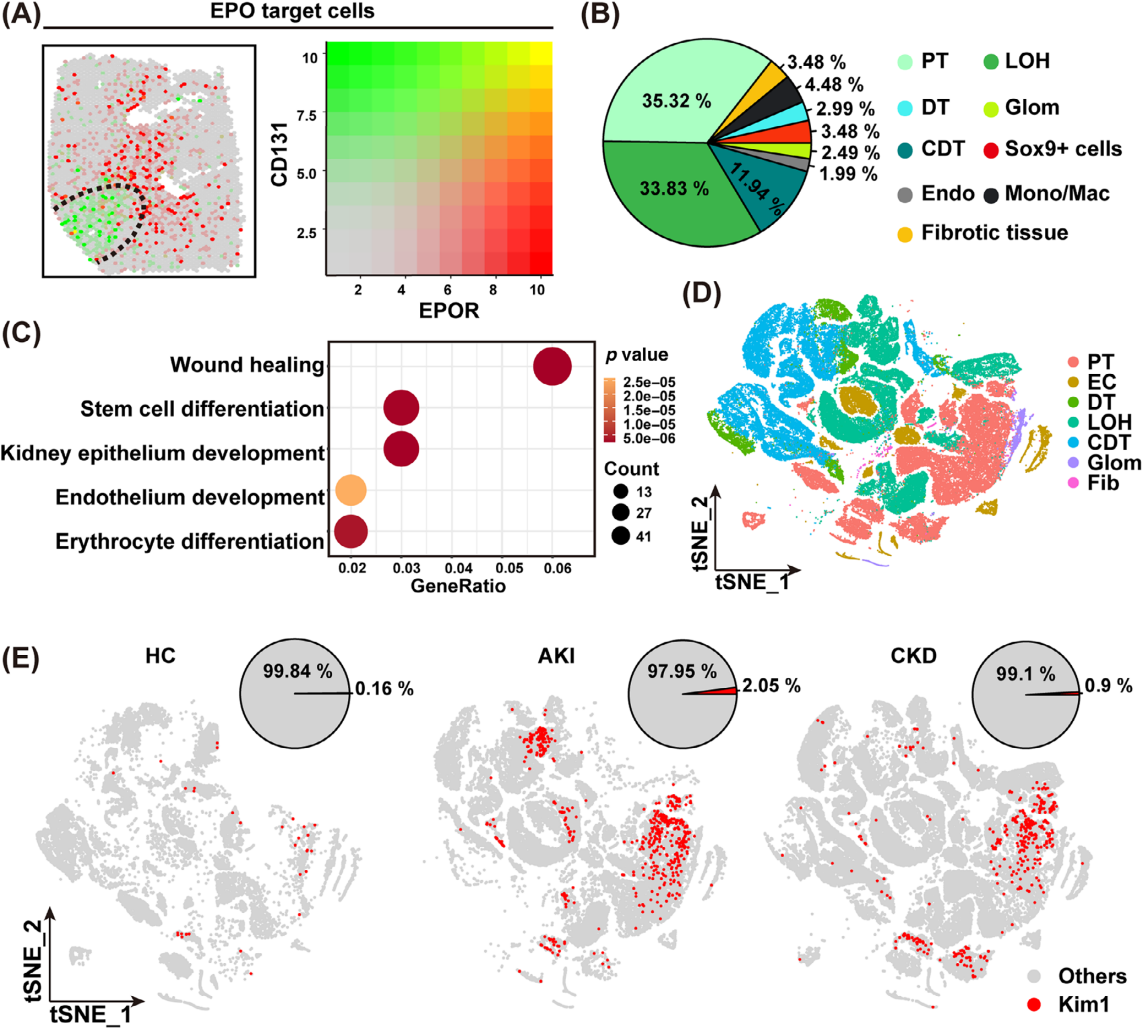
Spatial and functional analysis of EPO‐targeted cells in kidney tissue and injury models. (A) Spatial expression distributions of EPOR (red) and CD131 (green) within the tissue, as well as their coexpression patterns (left). Color saturation reflects gene expression strength, with deeper colors indicating higher expression levels (right). (B) Identification and proportions of cell types targeted by EPO, characterized by EPOR/CD131 coexpression. (C) Gene Ontology enrichment analysis of differentially expressed genes in EPO‐targeted cells and nontargeted cells in the injury model (*p *< 0.05). (D and E) t‐SNE visualization of kidney cell subsets from HC (*n* = 20), AKI (*n* = 12), and CKD (*n* = 17), with visualization and quantification of Kim1 gene expression (GSE183276).

We also tried to identify the potential target cells or tissues for EPO in the mouse kidney. At the molecular level, the tissue‐protective effect of EPO was known to be mediated by the EPO receptor (EPOR) associated with the CD131 subunit [24]. So here we analyzed the spatial distribution of EPOR+ single positive as well as the EPOR+CD131+ double positive tissue in kidney spatial transcriptomic data. The data showed that the EPOR+ tissues were widely distributed within the kidney. In contrast, the EPOR+/CD131+ tissues were concentrated in the injured area of the kidney, or the areas adjacent to the injury area, suggesting their potential role in injury protection or repair (Figure 3A). Interestingly, we observed that the putative EPO target tissues were predominantly proximal tubules or Loop of Henle (Figure 3B). Gene ontology (GO) analysis revealed that EPO target tissues expressed genes whose function were related to “wound healing,” “kidney epithelium development,” “endothelium development,” and “stem cell differentiation” (Figure 3C). Altogether these findings suggested a possible mechanism that local supplement of EPO could contribute to kidney protection by activating the wound healing process in proximal tubules/Loop of Henle.

### 2.2 Engineering and Intrarenal Implantation of Injury‐Responsive EPO‐Producing Cells

**FIGURE 4:**
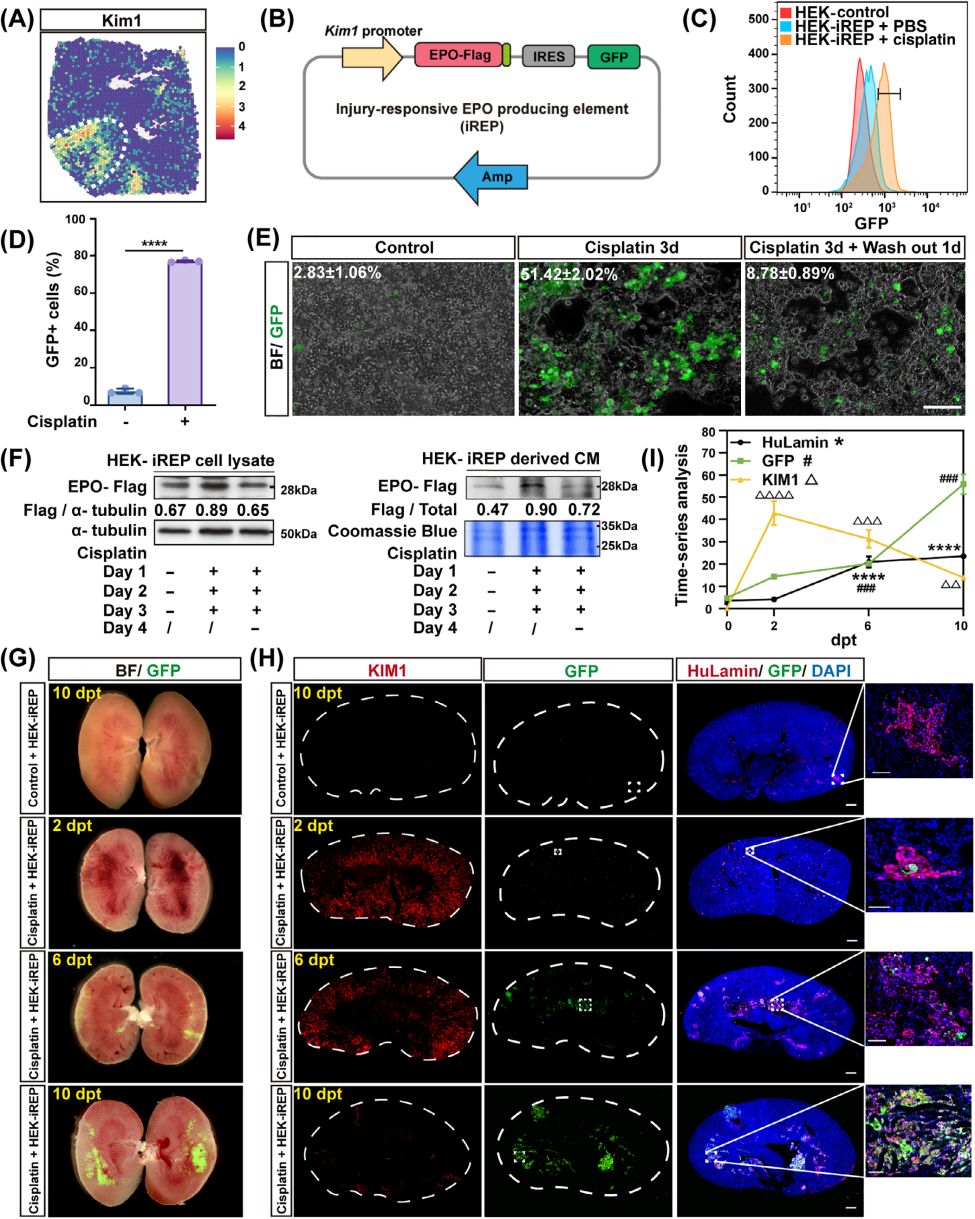
Engraftment and activation of HEK–iREP cells. (A) Expression pattern of Kim1 gene in the mouse injury model. (B) Schematic diagram of the injury‐responsive EPO‐producing (iREP) element design. (C and D) Gene induction in HEK–iREP cells with the GFP reporter. Representative flow cytometry profiles of GFP expression (C) and the percentages of GFP+ cells (D) were shown. *n* = 3 biological replicates in each group. (E) HEK–iREP cells harboring the GFP reporter, were exposed to 10 µg/mL cisplatin for 3 days and subsequently washout for 1 day, visualized through bright‐field and fluorescent images. Scale bar = 50 µm. *n* = 3 biological replicates in each group. (F) Detection of intracellular and extracellular (conditional medium, CM) EPO‐Flag expression, normalized to α‐tubulin. Coomassie blue staining was shown to demonstrate equivalent loading in CM samples. (G) Time‐series observations of HEK–iREP cell intrarenal transplantation by stereomicroscope. Kidneys were longitudinally midline‐opened for direct GFP visualization. dpt, days posttransplantation. (H) Immunostaining for HuLamin and GFP of the kidney after HEK–iREP cell transplantation. The white dashed line indicated the outline of the kidneys. dpt, days posttransplantation. HuLamin: Lamin A + C, a human‐specific nuclei antigen. Scale bar = 200 µm, 50 µm for magnified views. (I) Quantification of marker expressions corresponding to the images in panel H. *n* = 3 biological replicates in each group. Each group consisted of five mice. Data are shown as mean ± SD. ^△△^ *p* < 0.01, ^△△△^ or ^###^ *p* < 0.001, ^△△△△^ *p* < 0.0001, ****p < 0.0001 versus control + HEK–iREP.

Since we proved that a local supplement of EPO was beneficial for injured kidneys, we proceeded to design a novel system that could release EPO spontaneously in response to the kidney injury. The Kim1 gene, known as a specific and sensitive marker for kidney injury, is highly expressed in renal epithelial cells during both acute [25, 26] and chronic kidney injuries [27]. It is also considered as a prognosis marker of multiple kidney diseases [28]. Based on the single‐cell transcriptome analysis of human kidneys accessed through the public database (GSE183276), we confirmed an increased number of Kim1+ cells in patients with either acute kidney injury (13‐fold increase) or CKDs (6‐fold increase) (Figure 3D,E). Our mouse kidney transcriptomic data further confirmed expression of Kim1 mRNA specifically in the injured area (Figure 4A). And Kim1 expression was detected across various renal tubular cell types (Figure S2A). Additionally, a subset of Kim1+ cells exhibited coexpression of Sox9, along with proliferative capacity (Figure S2B). Therefore, to sense acute and chronic kidney injury, we generated a lentiviral construct enabling the expression of target genes (including flag tagged murine EPO and IRES–GFP) under the control of a human Kim1 gene promoter (Figure 4B).

In order to examine the function of the Kim1 promoter‐driven, injury‐responsive EPO production (iREP) construct, we transfected the iREP into the human HEK–293T cells (HEK–iREP). HEK–293T cells transfected with Kim1–IRES–GFP alone (without EPO) served as the control (HEK–control). HEK–293T cells were used because of their embryonic kidney origin and are widely used for efficient protein production in vitro. After exposing the engineered HEK–293T cells to 10 µg/mL cisplatin for 3 days in vitro, we found that this renal toxic drug induced GFP expression in an average of 77.1% of HEK–iREP cells, in comparison with a 7.4% baseline observed in control HEK cells treated with PBS (Figure 4C,D). Of note, when cisplatin was withdrawn for 1 day after the 3‐day cisplatin treatment, GFP expression decreased to 17% of the peak level (Figure 4E), suggesting that the iREP effect could be automatically “switched down.” A similar effect was confirmed by Western blot analysis of EPO‐Flag expression. We also confirmed that both intracellular and secreted EPO levels exhibited a similar dynamic pattern (Figure 4F). These results indicated that the iREP‐engineered HEK–293T cells were functional in vitro.

To examine the function of HEK–iREP in vivo, we implanted the engineered cells into the kidneys of immune‐deficient NOD‐SCID mice. Total 8 × 10^6^ HEK–iREP cells were implanted into either healthy or cisplatin‐induced acutely injured kidneys (Figure 4G). 10 days posttransplantation (dpt), some HEK–iREP cells engrafted into the healthy kidneys, as shown by immunostaining of the human‐specific Lamin antigen. However, very little GFP signal could be detected in the healthy kidney, indicating a basal level of iREP activation. In contrast, the cisplatin‐injured kidney displays more engraftment of HEK–iREP cells, as well as a much more prominent GFP signal than that in the healthy kidneys (Figure 4H). Time‐course analysis revealed peak KIM1 expression at 2 dpt and lasted to 10 dpt. Lagging behind the KIM1 change, the GFP signal was detectable at 6 dpt and reached a much higher level at 10 dpt (Figure 4I). Consistently, the renal tubular injury score also reached its maximum at 2 dpt and persisted through 10 dpt (Figure S3A). These data suggested that the iREP could be rapidly activated in response to kidney injury in vivo.

### 2.3 Intrarenal Implantation of HEK–iREP Cells Ameliorated Both Anemia and Kidney Injury

**FIGURE 5:**
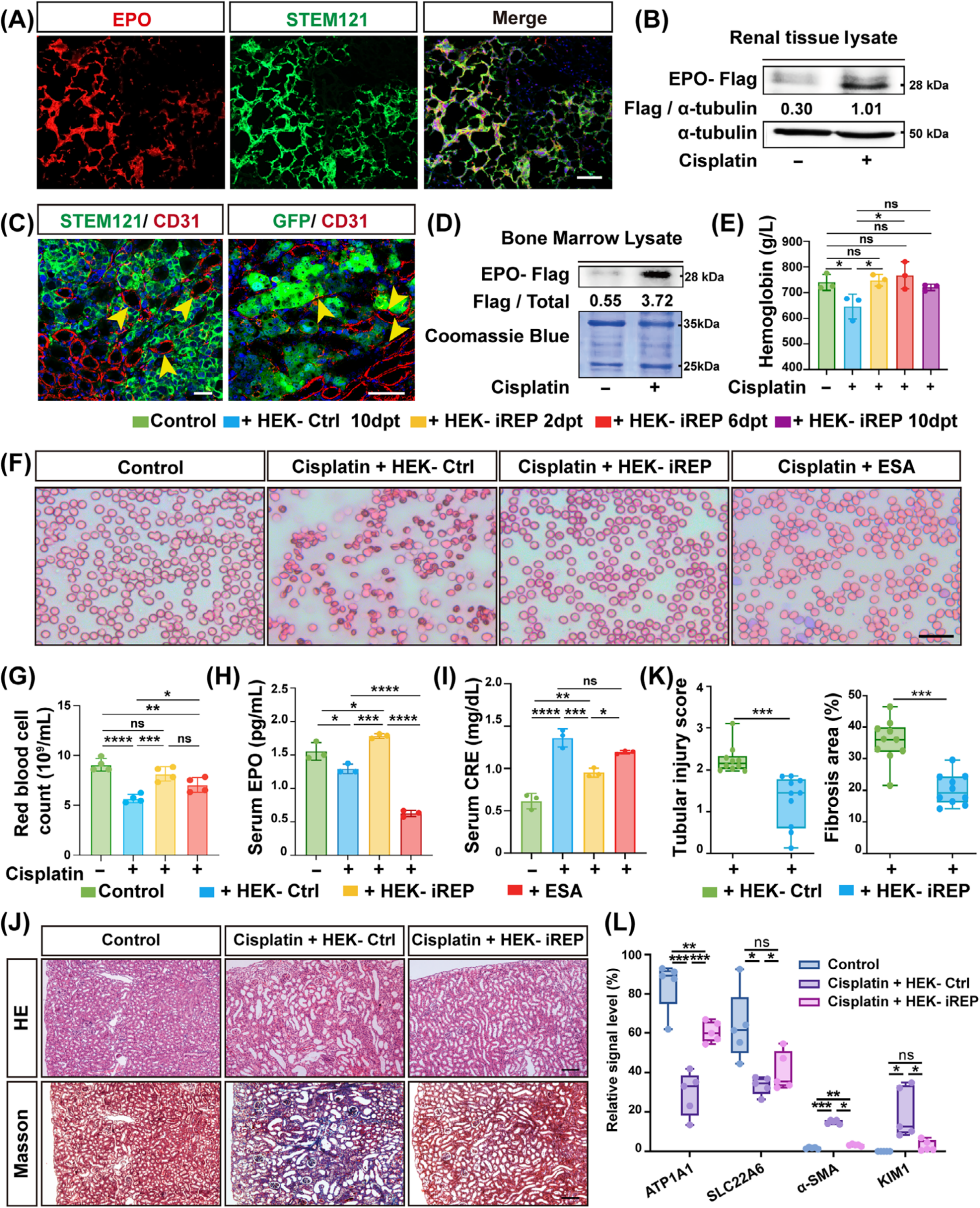
Function of intrarenal implanted HEK–iREP cells in cisplatin‐injured mice. (A) Immunofluorescence of EPO expression in the engrafted HEK–iREP cells 6 days after intrarenal transplantation. STEM121, a human‐specific antigen. Scale bar = 100 µm. (B) Western blot illustrating EPO‐Flag expression levels in healthy and cisplatin‐injured kidney tissues. EPO‐Flag levels were normalized by α‐tubulin. (C) Paraffin‐embedded sections of the kidney 10 days after HEK–iREP cell transplantation, immunostained for CD31, GFP, and STEM121. Yellow arrows indicate STEM121+/ CD31+ or CD31+/ GFP+ cells. Scale bar = 100 µm. (D) Western blot illustrating EPO‐Flag expression in the bone marrow of healthy and cisplatin‐injured mice, accompanied by quantitative analysis. Coomassie blue staining ensures equal loading for bone marrow samples. (E) Time‐course analysis of hemoglobin levels. *n* = 3 biological replicates in each group per time point. (F) Representative images of red blood cells in each group. Scale bar = 20 µm. (G) Quantification of red blood cell counts at 10 dpt. *n* = 4 biological replicates in each group. (H) Serum EPO levels measured at 10 dpt. *n* = 3 biological replicates in each group. (I) Serum creatinine levels were measured at 10 dpt. *n* = 3 biological replicates in each group. (J) H&E and Masson's trichrome staining of the cisplatin‐injured mouse kidneys harvested at 10 dpt. Scale bar = 100 µm. (K) Quantitative evaluation of tubular injury scores and tubulointerstitial fibrosis based on H&E and Masson's trichrome staining. *n* = 10 different fields of view for statistical analysis. H&E and Masson's trichrome staining of the cisplatin‐injured mouse kidneys harvested at 10 dpt. Scale bar = 100 µm. (L) Quantification of fibrotic marker α‐SMA and proximal tubular markers ATP1A1 and SLC22A6 by Immunofluorescence at 10 dpt. *n* = 5 different fields of view for statistical analysis. dpt, days posttransplantation. HEK–Ctrl, HEK–control. Each group consisted of five mice. Data are shown as mean ± SD. *p < 0.05, **p < 0.01, ***p < 0.001, ns, no significant.

After 6 days of engraftment, we found that approximately 35% of the human cells (STEM121+) engrafted in the injured kidneys robustly express EPO (Figure 5A). Western blot analysis showed that the injured kidneys had much higher expression of EPO‐Flag than that in healthy kidneys (Figure 5B). Of note, we observed a large number of CD31+ capillary vessels surrounding engrafted GFP+ human cells (Figure 5C), suggesting the produced EPO by activated HEK–iREP cells could have entrance into the circulatory system. Consistently, we also detected higher EPO‐Flag expression in the bone marrow of injured mice (Figure 5D), suggesting that the produced EPO indeed entered the circulation to promote the hematopoietic process. Interestingly, although the iREP element was switched on by kidney injury, the engrafted HEK–iREP cells exhibited proliferative capability in vivo with minimal apoptotic marker expression after cisplatin treatment (Figure S3B).

Next, we examined the anemia correction and renal protection of engineered cells by implanting HEK–iREP cells into the left kidney of cisplatin‐injured mice, and compared their function with HEK–control cells. At 10 dpt, we observed that HEK–iREP cells completely rescued the hemoglobin (Hb) levels of injured mice to the normal level (Figure 5E). In terms of anemia correction, HEK–iREP cells significantly restored red blood cell counts compared with the HEK–control group (Figure 5F,G). Furthermore, HEK–iREP treatment led to a marked increase in murine EPO levels, an effect not observed in the ESA‐treated group (Figure 5H). Interestingly, endogenous EPO levels were even lower in the ESA group, possibly due to negative feedback suppression from the exogenous recombinant human EPO (rHuEPO) administration. In terms of kidney protection, HEK–iREP cells significantly decreased serum creatinine levels in mice compared with the HEK–control group (Figure 5I), whereas ESA treatment did not show a significant reduction in creatinine levels, consistent with previous findings. HEK–iREP cells markedly reduced the tubular injury score and renal fibrotic lesions (Figure 5J,K). Furthermore, data showed that HEK–iREP cells could improve the repair of renal tubules, as demonstrated by an expanded area of ATP1A1+ and SLC22A6+ staining compared with the HEK–control group. Additionally, the expression of the myofibroblast marker α‐SMA was much lower in the HEK–iREP group, suggesting fewer fibrotic lesions (Figures 5L and S3C).

In order to understand whether intrarenal engraftment was required for HEK–iREP activation, we performed subcutaneous implantation of HEK–iREP cells. The HEK–iREP cells engrafted beneath the skin failed to sense the cisplatin‐induced kidney injury (Figure S3D). In another experiment, the HEK–iREP cells were implanted into the mouse lungs through intratracheal injection following bleomycin‐induced pulmonary injury, and we observed activation of iREP as shown by prominent GFP signals in the mouse lung (Figure S3E). These results indicated that the HEK–iREP cells outside kidneys can respond to local injury signals from another organ, but are incapable of sensing a distant kidney injury signal.

### 2.4 Engineering SOX9+ Epithelial Cells to Gain Injury‐Responsive EPO Production Capacity

The above work based on HEK–293T cells suggested that the human embryonic kidney cells might have the potential to be a “universal” candidate cell for iREP engineering and kidney implantation. However, considering that allogenic cell implantation would undoubtedly face the immune rejection challenge and immortalized cells should raise potential risks for implantation, we aimed to identify alternative safer candidate cell types for engineering and autologous intrarenal implantation. Previous reports indicated the presence of immature SOX9+ epithelial cells in the adult human kidney tubules [29, 30]. Furthermore, mature tubular cells could undergo trans‐differentiation or de‐differentiation upon various injury stimuli and become new SOX9+ epithelial cells [19, 31]. These immature SOX9+ epithelial cells were believed to have the ability to further proliferate and differentiate into mature kidney tubular epithelial cells [32, 33].

**FIGURE 6:**
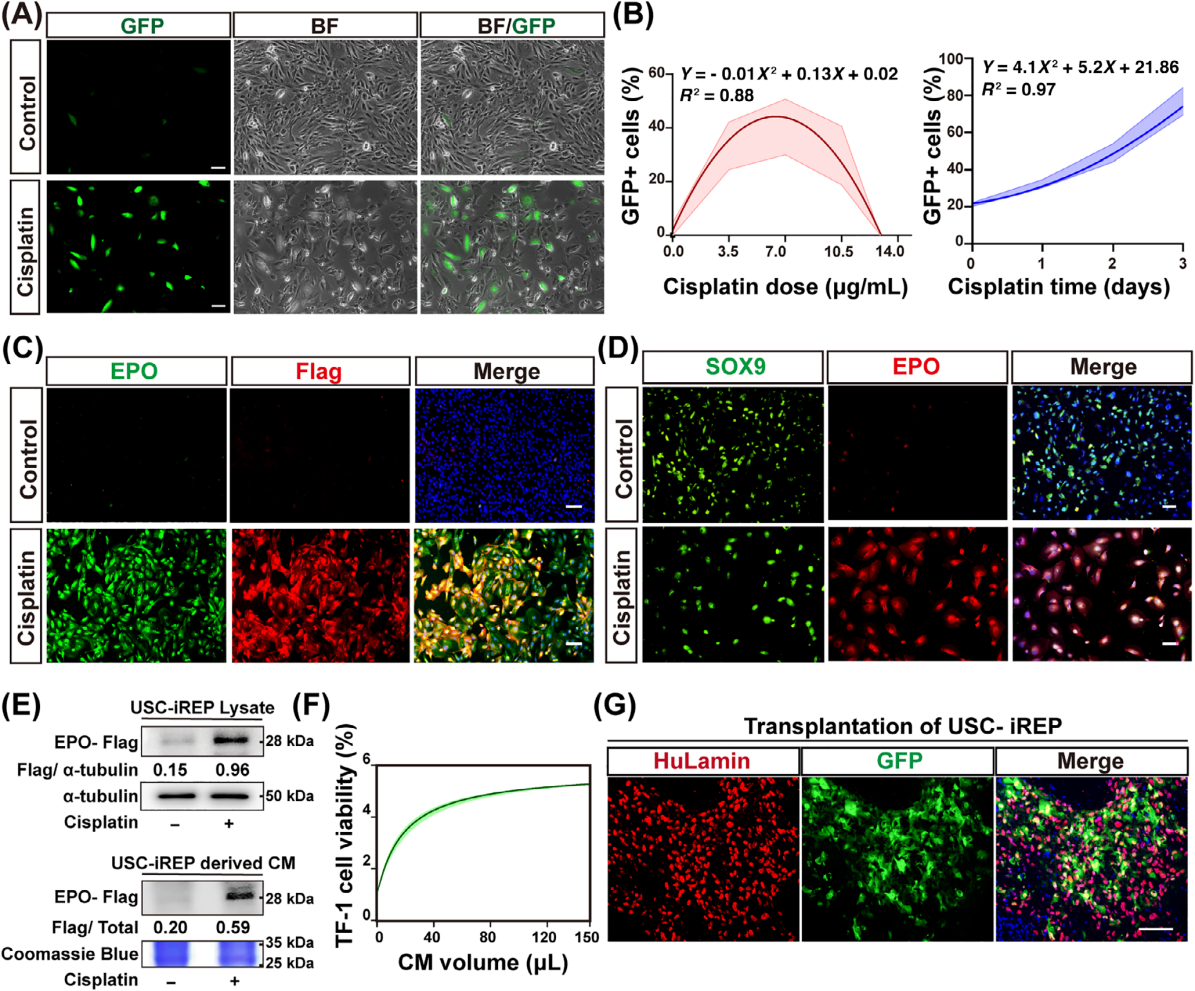
Intrarenal implantation of iREP‐engineered USCs and USC‐iREP activation. (A) USC‐iREP carrying the GFP reporter received 7 µg/mL cisplatin for 3 days. Bright‐field and corresponding fluorescent images were shown. *n* = 3 biological replicates in each group. Scale bar = 100 µm. (B) Response of USC‐iREP to cisplatin dosages (left) or time duration (right). The raw data were smoothed by the second‐order polynomial method, and curves representing the range from minimum to maximum. *n* = 5 different fields of view for statistics. (C) Immunofluorescence images of EPO and Flag in USC‐iREP after cisplatin stimulation. Scale bar = 100 µm. (D) Immunofluorescence images of SOX9 and EPO in USC‐iREP after cisplatin stimulation. Scale bar = 100 µm. (E) Western blot analysis of USC‐iREP cell lysate and CM, normalized to α‐tubulin, with Coomassie blue staining ensuring equal loading for CM samples. (F) TF‐1 human erythroleukemia cells were stimulated with CM from USC‐control or USC‐iREP at indicated concentrations. The TF‐1 cell viability was evaluated by CCK8 assay. *n* = 4 biological replicates in each group. (G) Representative immunostaining image of USC‐iREP intrarenal transplantation showing GFP and HuLamin expression. The activated USC‐iREP exhibited GFP positivity. Scale bar = 50 µm. HuLamin, Lamin A + C, a human‐specific nuclei antigen. Each group consisted of five mice.

We next transduced the urine‐derived SOX9+ epithelial cells (USCs) derived from healthy donors with iREP‐expressing lentivirus as described before. For activation of USC‐iREP, the engineered cells were treated with 7 µg/mL cisplatin for 3 days. The results showed that cisplatin‐induced increased GFP expression (Figure 6A). To quantify the relationship between the injury level and the iREP activation in USCs, we treated the cells with various cisplatin dosages ranging from 0 to 14 µg/mL. The result showed that the GFP+ cell ratio peaked at 7 µg/mL cisplatin treatment, while a lower cisplatin level could not fully activate the Kim1 promoter, and a higher cisplatin level would lead to cell death. A mathematical model was generated to show the relationship between cisplatin concentration (*x*) and activated cell ratio (*y*) as *y* = −0.01*x* ^2^ + 0.13*x* + 0.02. Also, the ratio of activated cells (*y*) was positively correlated to the time duration treated by cisplatin (*x*) following a mathematical model: *y* = 4.1*x* ^2^ + 5.2*x* + 21.86 (Figure 6B).

Consistently, immunostaining showed that a 72 hours cisplatin administration induced human EPO‐Flag expression in more than 80% of the USC‐iREP, in contrast to a background expression in only 3% of USC‐iREP free from cisplatin treatment (Figure 6C). Immunostaining further showed that EPO was coexpressed with SOX9+ cells (Figure 6D). Similar results were confirmed by western blot using whole cell lysates or conditional medium (Figure 6E). Of note, the secreted human EPO derived from USC‐iREP supernatant was able to stimulate the proliferation of human erythroleukemia cell TF‐1 in a dose‐dependent manner (Figure 6F). Transplantation of the USC‐iREP into cisplatin‐injured kidney led to activation of GFP signal in vivo (Figure 6G), although the signal intensity was lower than that in HEK–iREP cells. Altogether these findings indicated that kidney injury signals could induce functional human EPO synthesis and secretion in USC‐iREP, making it an alternative candidate other than the HEK–iREP cells.

## 3 Discussion

The kidney, known as an important endocrine organ [34], secretes multiple hormones including PGE2 [35], renin [36], and EPO [37]. EPO is widely recognized as a hormone that promotes the red blood cell production. However, the protective function of EPO in the kidneys is still a subject of debate. Previous reports revealed that treatment with even a single high dose of human EPO or its analogs administration could ameliorate acute kidney dysfunction by reducing apoptotic cell death and increasing local NO bioavailability [38]. Meanwhile, low‐dose EPO treatment provided renal endothelial protection and rescued vascular sclerosis in 5/6 nephrectomy‐injured rats through the activation of the prosurvival Akt signaling pathway [39]. Although most preclinical studies in animals suggest that EPO could alleviate inflammatory damage and fibrosis progression in the kidneys, clinical observations have not demonstrated a protective effect of EPO on acute kidney injury [40]. In our study, we found that the renal protective function of EPO was at least partially determined by its local concentration in the kidney. Additionally, infection with lentiviral vectors cannot be restricted to a specific site and may potentially lead to systemic dissemination. Therefore, here we tried to use intrarenal EPO‐producing cell implantation to enhance EPO local concentration.

Implantation of hormone‐producing cells has been extensively explored and even tested in clinical trials in the field of endocrinology. For example, by subcutaneous implantation of pancreatic endoderm cells, C‐peptide can be released in a glucose‐responsive manner in patients with type 1 diabetes [41, 42, 43, 44]. In the EPO field, previous work demonstrated EPO‐gene enhanced mesenchymal stem cells (MSCs) or kidney‐derived MSCs with sustained EPO‐producing capacity [45, 46, 47]. The engineered MSCs showed improved antiapoptosis and renal protective effects than original MSCs by intraperitoneal injection [48, 49, 50]. However, these studies were based on a constitutive EPO production system, which would inevitably lead to excessive EPO accumulation in circulation [51, 52], and raise potential risk issues such as high blood viscosity and even thrombosis. Other earlier studies used doxycycline to control EPO production and secretion in implanted engineered primary myoblasts in mice [11], which required manual monitoring of the red blood cell count and kidney status to decide the doxycycline level. In comparison, our current work aiming to achieve automatic, local release of native EPO hormone under the control of renal injury‐responsive gene promoter might be considered a “smarter” version of engineered cells.

The present study has some limitations. First, the research conducted so far is based on an AKI model in mice. Although it is known that the activation of the Kim1 signaling pathway occurs both in AKI and CKD, the continuous impact of chronic kidney damage on the iREP activation remained unclear. Second, the data obtained so far indicated that moderate kidney injury could enhance the expression levels of EPO; however, excessive kidney injury signals may lead to apoptosis of the engineered cells, resulting in a decrease in the overall expression levels of EPO. To address this issue, we are currently exploring other cell engineering strategies such as overexpression of BCL2 gene [53] against cell apoptosis. Finally, the long‐term effect of HEK–iREP cell or USC‐iREP implantation in the kidney has not been carefully investigated in current work. If the above limitation could be properly addressed in the future, the implantation of genetically engineered cells could become a promising approach for the automatic delivery of EPO and other bioactive molecules, allowing alleviation of anemia and kidney injury.

In this study, we developed a renal injury‐responsive cell implantation strategy for localized EPO delivery to enhance kidney protection while minimizing systemic side effects. Although challenges related to chronic injury responses and long‐term cell viability remain, this proof‐of‐concept lays the groundwork for future smart cell‐based therapies.

## 4 Materials and Methods

### 4.1 Mouse Kidney Injury Models

The 6∼8‐week‐old male C57BL/6J mice and immune‐deficient NOD‐SCID mice were purchased from Shanghai SLAC Laboratory Animal Center, China. To establish the mouse trauma model, mice were anesthetized, an incision was made in the left lateral peritoneum to expose the left kidney, and the renal artery was clamped with hemostatic forceps. Then, a straight incision was made on the back of the kidney with a surgical blade to create a trauma injury. After removing any excess blood, the incision was sealed with FuAiLe Medical glue. The hemostatic forceps were then removed from the renal artery. The muscle layer was closed with sutures (Ethicon, Germany), and the skin was closed as the final step.

For the cisplatin‐induced renal injury and anemia model, male C57BL/6J mice were injected intraperitoneally with a single dose of 10 mg/kg cisplatin. The survival rates and weights of injured mice were monitored. Kidney tissues and blood samples were harvested at specified time points for further analysis.

### 4.2 Human Samples

Human urine samples were collected from healthy volunteers. All the samples were obtained following clinical standard operating procedure under the patient's consent and approved by the Shanghai Tongji Hospital stem cell clinical research ethics committee [2021] GXB‐04. Written informed consent was obtained from all participants.

### 4.3 USCs Isolation

Human USCs were generated from urine samples and expanded by manual work as previously described [54].

### 4.4 Construction of iREP Vector

The pHIV‐GFP and pHIV‐Luciferase plasmids were purchased from Addgene (USA). The sequences of the constructed plasmids were verified by Sanger sequencing (Beijing Genomics Institute, China).

For the generation of the iREP system, an EPO‐Flag fragment from mice or humans was synthesized by Beijing Genomics Institute (China). The sequences of the constructed plasmids were verified by Sanger sequencing (Beijing Genomics Institute, China).

### 4.5 Generation of iREP‐Engineered Cells

The human embryonic kidney epithelial cell line HEK–293T (HEK) was obtained from the ATCC (USA). HEK cells were cultured and propagated in Dulbecco's modified Eagle's medium (Gibco, USA) supplemented with 10% FBS (ExCell Bio, China) and 1% P/S (Gibco, China). To generate EPO‐producing cells, HEK cells were transfected with prepared lentiviral supernatant for 48 h according to the manufacturer's protocol (Beyotime Biotechnology, China).

USCs from healthy donors were expanded and prepared for iREP transduction. The generation of USC‐iREP was performed by a lentiviral system for 48 h. Then, the USC‐iREP was expanded for further experiments.

### 4.6 In Vitro Functional Assay of iREP‐Engineered Cells

The iREP‐engineered cells were seeded and treated with various cisplatin dosages and duration time. Cells were monitored and images were acquired to obtain the induced GFP reporter signals.

The proliferation of TF‐1 cells (Procell, China) treated with conditional medium (CM) samples was evaluated. Following CM treatment, the cells were incubated with a CCK8 kit (Dojindo, Japan), and the optical density at 450 nm was measured.

### 4.7 Intrarenal Transplantation

For intrarenal transplantation, 8‐week‐old NOD‐SCID mice were damaged by 7 mg/kg cisplatin 4 days before implantation. Under stable anesthesia, the fur above the left kidney was shaved, and a sterile surgical procedure was performed to expose the kidney. Approximately 8 × 10^6^ HEK–control or HEK–iREP cells were resuspended in 40 µL of PBS. Cell intrarenal transplantation was performed using a microliter syringe, targeting the cortical region of the injured kidney. Mice were sacrificed at different time points after the transplantation. Kidney tissues, blood, and bone marrow samples were collected for further analysis.

### 4.8 Heterotopic Transplantation

For subcutaneous implantation, 8‐week‐old NOD‐SCID mice were damaged by 7 mg/kg cisplatin 4 days before implantation. Approximately 5 × 10^6^ HEK–iREP cells were injected for each mouse. Mice were sacrificed 7 days after the transplantation and skin samples were harvested for further analysis.

The mouse lung was injured by intratracheally instilling with 6 U/kg body weight of bleomycin (Selleckchem, USA) 7 days before transplantation. Then, mice were anesthetized by isoflurane and rested on a stand. 3 × 10^6^ HEK–iREP cells were suspended in 50 µL of PBS for transplantation. Intratracheal aspiration was performed by injecting the cells into the trachea via the mouth. Mice were sacrificed 3 days after the transplantation. Bright and direct fluorescence images of the transplanted lung were acquired under the fluorescence stereomicroscope (MVX10; Olympus, Japan).

### 4.9 ESA Treatment

8‐week‐old NOD‐SCID mice were induced with acute kidney injury by a single dose of cisplatin (7 mg/kg). Four days after injury, the mice were injected with rHuEPO (SEPO, China) at a dose of 100 IU/kg every other day. A total of three injections were administered. At 10 days posttreatment (10 dpt), the mice were sacrificed, and blood samples were collected for hematological analysis.

### 4.10 Histology and Immunofluorescence

H&E, Masson's trichrome, and immunofluorescence staining were performed according to standard protocol. Histological images were visualized and captured with an Olympus IX73 microscope and a Leica microscope. Quantitative image analyses were performed using the ImageJ software. Tubular injury scoring was determined as follows: 0, 0–5%; 1, 5–10%; 2, 11–25%; 3, 26–45%; 4, 46–75%; 5, >76% [54].

### 4.11 Blood Sample Analysis

The blood samples were taken by suborbital vein and serum was separated by centrifugation. The blood samples with anticoagulant were prepared for blood cell smears, red blood cells, and reticulocyte counting. The morphology of red blood cells was identified by Reichsen‐Giemsa staining (Yeasen, China), and the number of reticulocytes was analyzed by Brilliant Cresyl Blue staining (Solarbio, China). The whole blood Hb was measured by a mouse ELISA kit (COIBO BIO, China). The serum samples were used to detect the level of creatinine (BioAssay Systems, USA) and EPO (Solarbio) in mice.

### 4.12 Western Blot Analysis

All preprocessed samples were lysed in RIPA buffer supplemented with PMSF (MCE, USA) followed by standard Western blot procedure [55]. The immunoblots were blocked and incubated with either anti‐Flag (1:1000; Sigma, Germany) or anti‐α‐tubulin antibody (1:3000; Abways Technology, China). After incubation with the HRP‐conjugated secondary antibody (1:3000; Beyotime Biotechnology), the PVDF membranes were visualized with an enhanced chemiluminescence system kit (Beyotime Biotechnology), and the grayscale of the bands was analyzed by ImageJ [56].

### 4.13 Single‐Cell RNA Sequencing Data Analysis

Public datasets (GSE183276 and GSE139107) were used for bioinformatics analysis. Seurat (version 4.3.0.1) was used to read a combined gene‐barcode matrix of all samples. After merging the highly variable genes, principal component analysis was obtained by identifying the top 2000 variable genes from each dataset with FindVariableFeatures and merging the list; then principal component analysis (“RunPCA” function) was performed. Clustering and tSNE were performed in Seurat using the “PCA” data type as the dimensional reduction type.

### 4.14 Spatial Transcriptome Analysis

6.5 × 6.5 mm fresh tissues were concurrently frozen and embedded in the optical cutting tissue compound in liquid nitrogen and delivered to LC‐Bio Technologies (Hangzhou, China) in dry ice for spatial transcriptome sequencing.

The Seurat package was used to perform gene expression normalization, dimensionality reduction, spot clustering, and differential expression analysis. Briefly, spots were filtered for a minimum detected gene count of 100 genes. Normalization across spots was performed with the SCTransform function and 3000 highly variable genes were selected for principal component analysis. For spot clustering, the first 30 PCs were used to build a graph, which was segmented with a resolution of 0.8. Wilcox algorithm was used to perform differential gene expression analysis for each cluster via the FindAllMarkers function.

### 4.15 Bioinformatic Analysis

GO biological process and pathway enrichment analyses of differentially expressed genes in sc‐RNA‐seq data were performed using Metascape (Ver. 3.5, http://metascape.org) or implemented by the ClusterProfiler R package, and the results were visualized with the ggplot2 R package. GO terms with a *p* value less than 0.05 were considered significantly enriched by differentially expressed genes.

### 4.16 Mathematical Modeling and Statistical Analysis

Statistical analyses were performed using Graphpad Prism software (GraphPad 9.5.1). To visualize the relationship between cisplatin stimulation and activation of iREP, a scatter plot and a quadratic function (*y* = *ax* ^2^ + *bx* + *c*) were generated to fit the data to yield a polynomial curve. Results were expressed as mean ± SD. Unpaired results were assessed by ordinary one‐way ANOVA. Comparisons among multiple groups were analyzed using Tukey's multiple comparison test. Differences with *p* ≤ 0.05 were considered statistically significant.

## Author Contributions

W.Z. and T.Z. designed the study. H.N., C.L., Y.X.H., S.Y.W., X.P.Z., J.D., and Z.X.Z. performed the experiments. W.Z., H.N., C.L., Y.X.H., and S.Y.W. contributed to the data analysis and interpretation. J.L.H., C.Y., and Y.J.W. contributed to the clinical sample acquisition. W.Z. and T.Z. secured the funding for the study. The manuscript was written by H.N., C.L., and Y.X.H. with revisions made by W.Z., T.Z., Y.J.W., and C.Y. All authors have read and approved the final manuscript.

## Conflicts of Interest

Authors Wei Zuo, Ting Zhang, and Yujia Wang are employees of Regend Therapeutics Co., Ltd., but have no potential relevant financial or nonfinancial interests to disclose. The other authors declare no conflicts of interest.

## Ethics Statement

All animal experiments were carried out following Chinese National Guidelines GB/T 35892–20,181, as well as under the guidance of the Institutional Animal Care and Use Committee of Tongji University.

## Supporting information




**Figure S1**: Function of recombinant EPO treatment. **Figure S2**: Transcriptomic profiling of KIM1 expression. **Figure S3**: Transplantation of HEK‐iREP cells.

## Data Availability

RNA‐seq data generated during this study have been deposited at GEO under accession codes GSE268847. The majority of the analysis was carried out using published and freely available software and pre‐existing packages mentioned in the methods. More detailed materials and methods information is available in Supplementary information.
