# Supplementary material for: Implant Renal Injury‐Responsive Cells to Supplement Erythropoietin and Protect Kidney Injury
Source: MedComm (2020). 2025 Oct 30;6(11):e70438. doi: 10.1002/mco2.70438 (PMC12572954; doi:10.1002/mco2.70438)
Supplement: Supplementary file 1 — Figure S1: Function of recombinant EPO treatment. Figure S2: Transcriptomic profiling of KIM1 expression. Figure S3: Transplantation of HEK‐iREP cells. [file MCO2-6-e70438-s001.docx]

**Implant renal injury-responsive cells to supplement erythropoietin and protect kidney injury**

Hao Nie^1#^, Chen Liang^1#^, Yingxin He^1#^, Siyu Wang^1#^, Xiaopeng Zhang^1^, Jun Duan^1^, Jieli Huang^5^, Chen Yu^5^, Yujia Wang^1,3^, Zixian Zhao^1,4^, Wei Zuo^1,2,3^*, Ting Zhang^2,3^*

^1^State Key Laboratory of Cardiovascular Diseases and Medical Innovation Center, Shanghai East Hospital, School of Medicine, Tongji University, Shanghai, China

^2^Kiangnan Institute of Stem Cell, Hangzhou, China

^3^Super Organ R&D Center, Regend Therapeutics, Shanghai, China

^4^Department of Pharmacy, National University of Singapore, Singapore

^5^Department of Nephrology, Tongji Hospital, School of Medicine, Tongji University, Shanghai, China

*Corresponding authors:

Wei Zuo ([zuow@tongji.edu.cn](mailto:zuow@tongji.edu.cn))

Ting Zhang (winnie-tzhang@regend.cn)

#These authors contribute equally to this work.

**Method**

**Construction of iREP vector**

The pHIV-GFP and pHIV-Luciferase plasmids were purchased from Addgene (USA). To construct a Kim1 gene inducible vector, a plasmid encoding Kim1 promoter mRNA was generated by replacing the original EF-1α promoter with human Kim1 promoter cDNA in a lentiviral vector co-expressing luciferase and GFP reporters. The sequences of the constructed plasmids were verified by Sanger sequencing (Beijing Genomics Institute, China).

For the generation of the iREP system, an EPO-Flag fragment from mice or humans was synthesized by Beijing Genomics Institute (China). The plasmid encoding EPO-Flag mRNA was constructed by replacing luciferase fragment with mouse or human EPO-Flag cDNA in the Kim1 inducible lentiviral vector co-expressing GFP reporter. The sequences of the constructed plasmids were verified by Sanger sequencing (Beijing Genomics Institute, China).

To generate lentiviral particles, HEK-293T cells were co-transfected with the lentiviral expression vector and packaging construct to generate lentiviral supernatant.

**Generation of iREP-engineered cells**

The human embryonic kidney epithelial cell line HEK-293T (HEK), was obtained from the ATCC (USA). HEK cells were cultured and propagated in Dulbecco’s modified Eagle’s medium (DMEM, Gibco, USA) supplemented with 10% FBS (ExCell Bio, China) and 1% P/S (Gibco, China). To generate EPO-producing cells, HEK cells were transfected with prepared lentiviral supernatant for 48hr according to the manufacturer’s protocol (Beyotime Biotechnology, China).

USCs from healthy donors were expanded and prepared for iREP transduction. The generation of USC-iREP was performed by a lentiviral system for 48 hours. Then the USC-iREP was expanded for further experiments.

***In vitro* functional assay of iREP-engineered cells**

The iREP-engineered cells were seeded and treated with various cisplatin dosages and duration time. Cells were monitored and images were acquired to obtain the induced GFP reporter signals. The GFP expression levels were quantified by flow cytometry. The supernatant samples (conditional medium, CM) of iREP cells were centrifuged through Ultra-15 10K Centrifugal Filter Device (Amicon, Millipore) at 4,000 × g for 45 min at 4°C to collect concentrate whose mass was more than 10-kD.

The proliferation of TF-1 cells (Procell, China) treated with CM samples was evaluated. Following CM treatment, the cells were incubated with a CCK8 kit (Dojindo, Japan), and the optical density at 450 nm was measured.

**Histology and immunofluorescence**

Antibodies used for immunofluorescence include:

| Antibodies | Brand | Catalog No. | Working dilution |
| --- | --- | --- | --- |
| SOX9 | Abways | CY5400 | 1:200 |
| Ki67 | BD Pharmingen | 550609 | 1:100 |
| Caspase3 | CST | 9579S | 1:100 |
| SLC22A6 | Abcam | ab135924 | 1:200 |
| ATP1A1 | Santa Cruze | C464.6 | 1:200 |
| Lamin A/C | Abcam | ab108595 | 1:200 |
| GFP | Abcam | ab6673 | 1:500 |
| STEM121 | Sigma | Y40410 | 1:250 |
| EPO | Bioss | bs-2343R | 1:100 |
| CD31 | Proteintech | 280-1-AP | 1:200 |
| KIM1 | R&D | AF1817 | 1:100 |
| AQP1 | Abways Technology | CY6767 | 1:100 |
| Flag | Sigma | F1804-50UG | 1:200 |
| α-SMA | PROTEINTECH | 14395-1-AP | 1:200 |
| Alexa Fluor-conjugated Donkey 488/594 | Life Technologies | A-21206/ A-21207 | 1:200 |

**Western blot analysis**

To detect the EPO-Flag expression, cell supernatant samples were centrifuged through Ultra-15 10K Centrifugal Filter Device (Amicon, Millipore) at 4000 × g for 45 min at 4°C to collect concentrate whose mass was more than 10-kD. For kidney tissues or bone marrow suspension, samples were homogenized in lysis buffer with magnetic beads.

**Supplementary Figure**

**
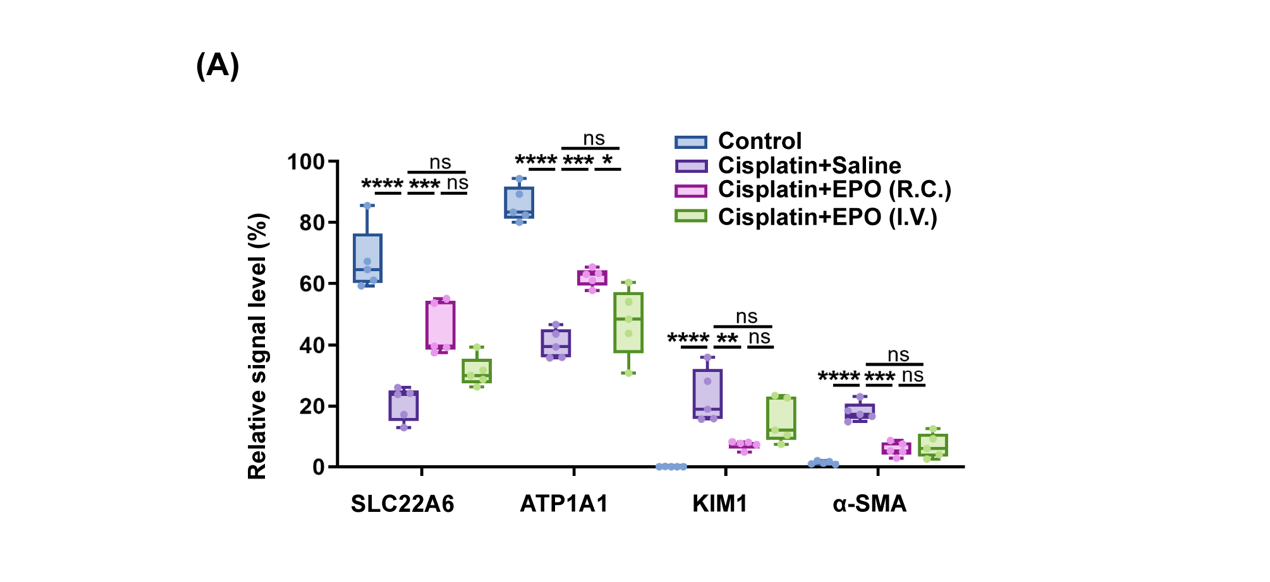
**

**FIGURE S1 | Function of recombinant EPO treatment.**

**(A)** Quantification of kidney injured marker (KIM1), fibrotic marker (α-SMA), and proximal tubular markers (ATP1A1 and SLC22A6) by Immunofluorescence at 10 days post-treatment. *n* = 5 different fields of view for statistics. Data were shown as mean ± SD. ** *P* < 0.01, *** *P* < 0.001, **** *P* < 0.0001, ns, no significant.


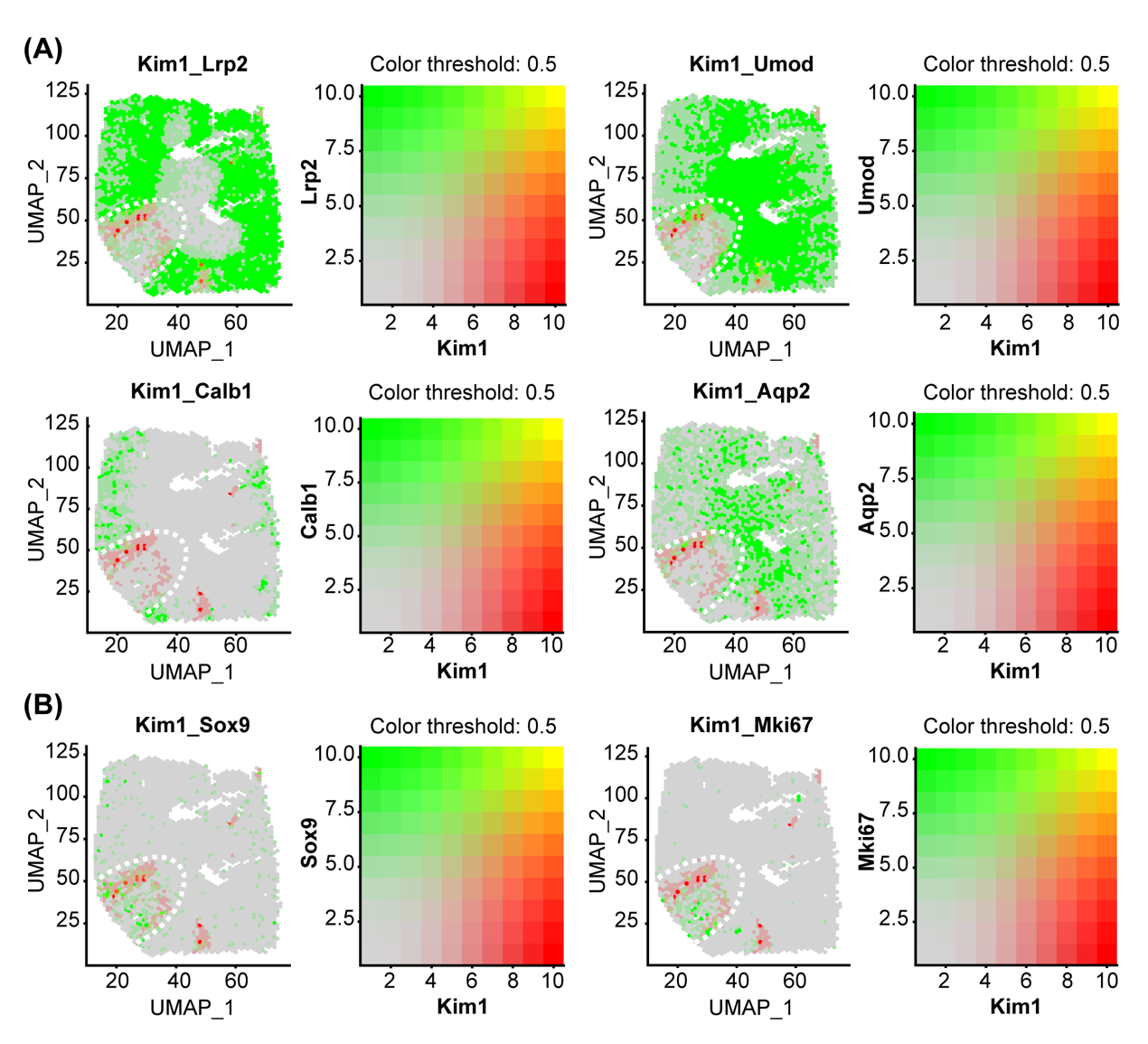


**FIGURE S2 | Transcriptomic profiling of KIM1 expression**

**(A)** Spatial expression patterns within tissues showing the distribution of different kidney epithelial tubule-related genes (Lrp2, Umod, Calb1, and Aqp2) in red, and Kim1 in green, along with their co-expression patterns. Color saturation reflects gene expression strength, with deeper colors indicating higher expression levels. **(B)** Spatial expression patterns within tissues show the distribution of Sox9 (left) or Mki67 (right) in red, and Kim1 in green, along with their co-expression patterns. Color saturation reflects gene expression strength, with deeper colors indicating higher expression levels.


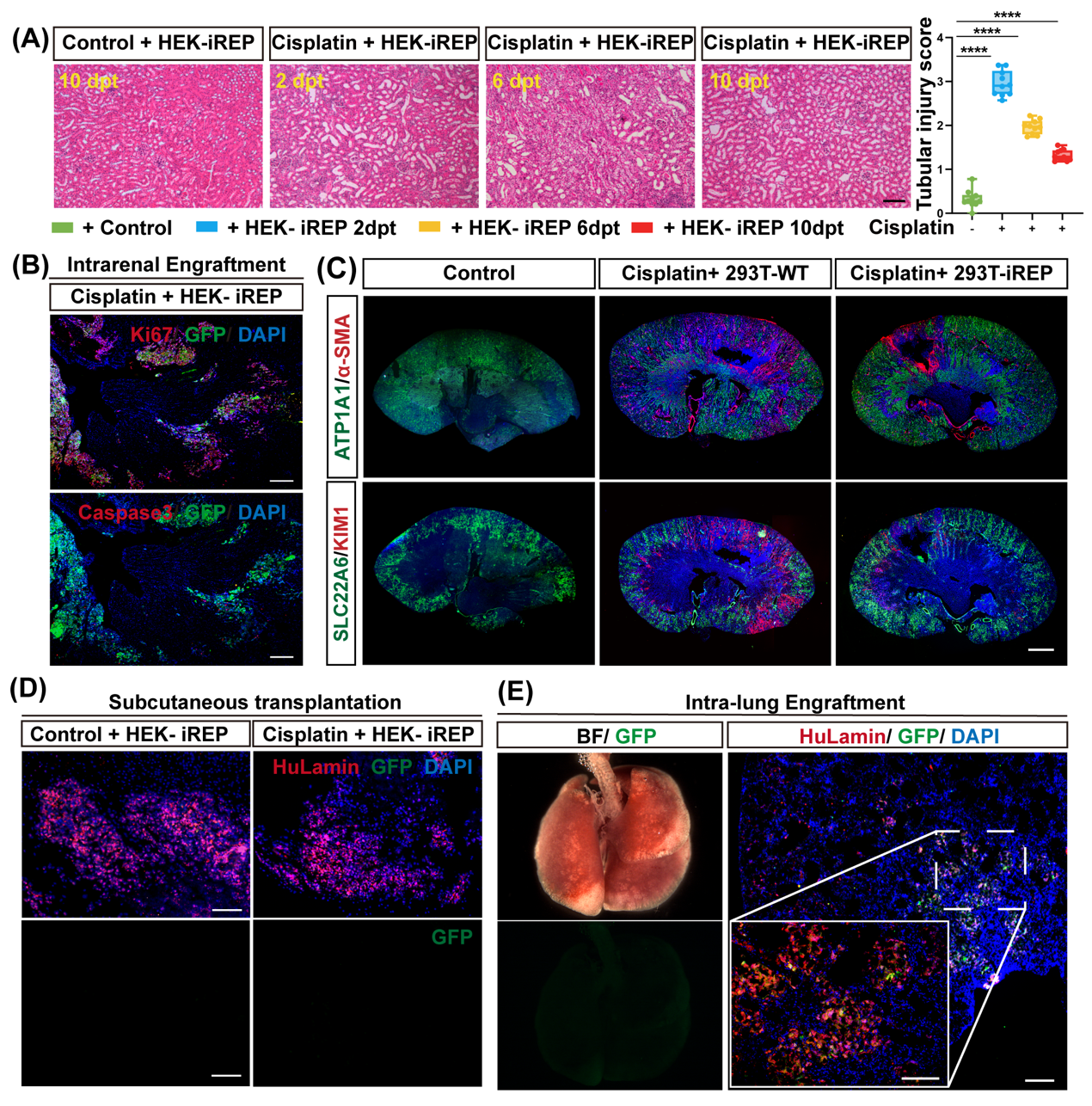


**FIGURE S3 | Transplantation of HEK-iREP cells.**

**(A)** H&E staining of time-series observations of HEK-iREP cell intrarenal transplantation (left). dpt, days post-transplantation. Scale bars = 100 μm. And Quantitative evaluation of tubular injury scores based on H&E staining (right). *n* = 10 different fields of view for statistical analysis. **(B)** Immunofluorescence analysis of proliferative (Ki67) and apoptotic (Caspase3) markers expression in engrafted HEK-iREP cells at 10 dpt. Scale bars = 100 μm. **(C)** Immunostaining of kidney sections showed expression of the renal injury marker (KIM1 and a-SMA) and renal tubule markers (ATP1A1 and SLC22A6) in the cisplatin-injured kidneys. Scale bar = 1 mm.

**(D)** Histological sections of cisplatin-injured mice skin after 7 days of subcutaneous engraftment of HEK-iREP cells, stained by HuLamin and GFP. HuLamin, Lamin A + C, a human-specific nuclei antigen. Scale bars = 50 μm. **(E)** Bright-field and direct fluorescence images of the bleomycin-injured lung 3 days after HEK-iREP cells intra-lung transplantation. Lung sections were subjected to immunostaining for HuLamin and GFP. HuLamin: Lamin A + C, a human-specific nuclei antigen. Scale bars = 100 μm, 50 μm for magnified view.
